# miRNA-130b is required for the ERK/FOXM1 pathway activation-mediated protective effects of isosorbide dinitrate against mesenchymal stem cell senescence induced by high glucose

**DOI:** 10.3892/ijmm.2014.1985

**Published:** 2014-10-29

**Authors:** JIANFENG XU, ZHEYONG HUANG, LI LIN, MINGQIANG FU, YANAN SONG, YUNLI SHEN, DAOYUAN REN, YANHUA GAO, YANGANG SU, YUNZENG ZOU, YUEGUANG CHEN, DADONG ZHANG, WEI HU, JUYING QIAN, JUNBO GE

**Affiliations:** 1 Department of Cardiology, Minhang Hospital, Ruijin Hospital Group, Shanghai Jiaotong University School of Medicine, Shanghai 201199, P.R. China; 2 Department of Cardiology, Zhongshan Hospital, Fudan University, Shanghai 200032, P.R. China; 3 Institutes of Biomedical Scienses, Fudan University, Shanghai 200032, P.R. China; 4 Department of Cardiology, Eastern Hospital, Tongji University, Shanghai 200120, P.R. China

**Keywords:** mesenchymal stem cell, senescence, hyperglycemia, nitrate, mechanism

## Abstract

The present study was carried out to investigate the hypothesis that organic nitrates can attenuate the senescence of mesenchymal stem cells (MSCs), a superior cell source involved in the regeneration and repair of damaged tissue. MSCs were treated with high glucose (HG) in order to induce senescence, which was markedly attenuated by pre-treatment with isosorbide dinitrate (ISDN), a commonly used nitrate, as indicated by senescence-associated galactosidase (SA-β-gal) activity, p21 expression, as well as by the mRNA levels of DNA methyltransferase 1 (DNMT1) and differentiated embryo chondrocyte expressed gene 1 (DEC1), which are senescence-related biomarkers. It was also found that the senescent MSCs (induced by HG glucose) exhibited a marked downregulation in ERK activity and forkhead box M1 (FOXM1) expression, which was reversed by ISDN preconditioning. Of note, the inhibition of ERK phosphorylation or the downregulation of FOXM1 statistically abolished the favourable effects of ISDN. In addition, the investigation of the senescence-associated miR-130 family suggested that miR-130b mediates the beneficial effects of ISDN; it was found that the protective effects of ISDN against the senescence of MSCs were prominently reversed by the knockdown of miR-130b. Furthermore, the downregulation of ERK phosphorylation or FOXM1 expression decreased the miR-130b expression level; however, the suppression of miR-130b demonstrated no significant impact on ERK phosphorylation or FOXM1 expression. Taken together, to the best of our knowledge, the present study is the first to demonstrate the favourable effects of ISDN against HG-induced MSC senescence, which are mediated through the activation of the ERK/FOXM1 pathway and the upregulation of miR-130b.

## Introduction

Organic nitrates are a class of drugs that have been clinically used in the treatment of myocardial ischemia, as well as congestive heart failure for more than a hundred years. Nitrates release nitric oxide (NO), an endothelium-derived relaxing factor, executing a broad range of functions, including antioxidant, anti-inflammatory, antithrombotic and anti-atherogenic effects (1). Furthermore, NO has been shown to ameliorate the aging of cells (2), and small interfering RNA (siRNA) targeting endothelial NO synthase (eNOS) have been shown to reduce the anti-senescence effects of insulin (3). However, whether organic nitrates, a source of NO production, play a protective role against the senescence of bone marrow-derived mesenchymal stem cells (MSCs), which have potential for the repair of damaged tissue (4), remains to be clarified.

We have previously reported that MSCs are a superior stem cell source for cellular treatment and biomedical engineering due to their unique paracrine and immunosuppressive properties (5). However, cell aging, which can be induced by various pathophysiological states, including high glucose (HG), oxidative stress and inflammatory attack, can severely impair the therapeutic potential of MSCs in tissue regerenation (6). Therefore, the investigation of possible interventions to attenuate MSC senescence and the further investigation of the potential mechanisms involved is of critical clinical significance for patients with diabetes accompanied by organ injury, currently awaiting the regeneration of ‘viable’ MSCs (7,8).

The results of previous studies have led us to hypothesize that organic nitrates may prevent the onset of MSC senescence. Lauer *et al* (9) demonstrated that the lack of nitrite in the plasma was mainly responsible for the inability of exercise to reverse age-dependent endothelial dysfunction. Ward *et al* (10) also noted that the overexpression of eNOS, which results in endogenous NO production, boosted the capability of bone marrow cells, leading to a substantial improvement in left ventricular ejection fraction at 6 weeks. Accordingly, in the present study, we examined the effects of isosorbide dinitrate (ISDN), a commonly used organic nitrate, on the senescence of MSCs triggered by HG. The characteristics of cellular aging, such as increased expression of senescence-associated galactosidase (SA-β-gal), cell enlargement and the upregulation of p21 expression, were investigated. In addition, the mRNA expression of the typical senescence-associated cellular biomarkers (11), including DNA methyltransferase 1 (DNMT1) and differentiated embryo chondrocyte expressed gene 1 (DEC1) was detected to characterize growth arrest. We also investigated the underlying mechanisms responsible for the effects of ISDN based on the knowledge of the key roles of ERK phosphorylation (12) and forkhead box M1 (FOXM1) regulation (13) in cell cycle modulation, and further clarified the association between them. Considering that cellular senescence has recently been implicated in regulation of microRNAs (miRNAs or miRs) (14), crucial factors of diverse pathophysiological processes, we wished to explore the potential role of senescence-associated miRNAs in the effects of ISDN on MSC senescence.

## Materials and methods

All animal treatments were performed in strict accordance with the Guidelines for the Care and Use of Laboratory Animals published by the National Academy Press (NIH Publication no. 85–23, revised 1996). The study was approved by the Animal Care and Use Committee of Zhongshan Hospital, Fudan University.

### Isolation and culture of MSCs

Bone marrow-derived MSCs were purified from 4-week-old male Sprague-Dawley (SD) rats as previously described (15). Briefly, the bone marrow in the tibias and femurs was flushed using Dulbecco’s modified Eagle’s medium (DMEM; Gibco, Grand Island, NY, USA). Following incubation in DMEM supplemented with 10% fetal bovine serum (FBS; Gibco) at 37°C in an atmosphere of 5% CO_2_ for 24 h, the bone marrow solution was discarded, and the adherent cells were subsequently cultured. Subsequently, the spindle-shaped, adherent MSCs were isolated and expanded, and the culture medium was regularly changed every 3–4 days. Passage 4 MSCs, which were previously demonstrated to express typical MSC-related cell surface antigens (15), were used in the subsequent experiments.

### Experimental manipulation of MSCs

Native MSCs were starved for serum and glucose overnight and then exposed to medium with HG or ISDN (both from Sigma-Aldrich, St. Louis, MO, USA) at the indicated concentrations or mannitol (33 mM) for 24 h. The untreated MSCs served as the control group and those exposed to mannitol served as the negative control group. To examine the effects of ISDN on cell aging, the starved MSCs were treated with ISDN (50 μM) for 6 h prior to HG treatment. To explore the underlying mechanisms responsible for the effects of ISDN, the MSCs were incubated with PD98059 (ERK inhibitor, 20 μM; Santa Cruz Biotechnology Inc., Santa Cruz, CA, USA) or DMSO (dimethyl sulfoxide; Sigma-Aldrich) for 30 min prior to ISDN treatment or were subjected to FOXM1 knockdown using siRNA, followed by subsequent treatment with ISDN and HG.

### Western blot analysis

After the indicated treatments, the MSCs were lysed in RIPA lysis buffer containing 50 mM Tris-HCl (pH 7.4), 1% NP-40, 1 mM phenylmethylsulphonyl fluoride, 150 mM NaCl, 1 mM EDTA and 1% sodium deoxycholate. Protein lysates were separated by 10% SDS-PAGE and transferred onto polyvinylidene fluoride membranes. The membranes were washed 3 times with TBS (pH 7.6) buffer, soaked in 5% non-fat dry milk for 2 h and incubated overnight at 4°C with anti-p21 (#2947), anti-phosphorylated (p-)ERK (#4370) (both from Cell Signaling Technology, Danvers, MA, USA; both diluted 1:1,000) and anti-FOXM1 (sc-271746; Santa Cruz Biotechnology Inc.) polyclonal antibodies (diluted 1:300), followed by incubation with a horseradish peroxidase-conjugated secondary antibody (diluted 1:5,000) for 2 h at room temperature. The immune complexes were visualized using enhanced chemiluminescence detection reagents, and the band intensity was measured, quantified and analyzed using an Image System (Bio-Rad, Hercules, CA, USA). The β-actin (#8457; Cell Signaling Technology) band intensity served as the control for p21 and FOXM1 expression; similarly, total-ERK (#4695; Cell Signaling Technology) served as the control for p-ERK expression.

### Reverse transcription-quantitative (real-time) polymerase chain reaction (RT-qPCR)

Total RNA was isolated from the cultured MSCs using TRIzol reagent (Invitrogen, Carlsbad, CA, USA). Spectrophotometric OD_260_ measurements were used to determine the 2 μg RNA input for cDNA synthesis and cDNA was generated using a High Capacity cDNA Reverse Transcription kit (Applied Biosystems, Foster City, CA, USA) as recommended by the manufacturer. Real-time PCR was performed using the SYBR^®^ ExScript™ PCR kit (Takara Biochemicals, Kyoto, Japan) in a total volume of 10 μl in a Bio-Rad iQ5 optical module. The primer sequences used for the genes (*DNMT1*, *DEC1* and *β-actin*) were synthesized and are listed as follows: *DNMT1* forward, 5*′*-GTG AAG GAG AAA TTG AAT CTC TT-3*′* and reverse, 5*′*-GAG GAA GCT GCT AAG GAC-3*′*; *DEC1* forward, 5*′*-CCA GGA AAC CAT TGG ACT CAG -3*′* and reverse, 5*′*-AGA GGT CGG ATA CCA GCA TTT-3*′*; *β-actin* forward, 5*′*-CCA TTG AAC ACG GCA TTG-3*′* and reverse, 5*′*-TAC GAC CAG AGG CAT ACA-3*′*. The PCR amplification consisted of 40 cycles (95°C for 5 sec, 59.5°C for 30 sec) following an initial denaturation at 95°C for 30 sec. Melting curves were obtained at the end of the reaction by stepwise increases in temperature of 1°C/min from 59.5 to 95°C over a period of 35 min. The threshold cycle (Ct) value was defined as the fractional cycle number at which the fluorescence passed a fixed threshold. The fold -change in target mRNA expression was calculated using the 2^−ΔΔCt^ method following normalization to *β-actin* expression.

For microRNA detection, total RNA was extracted from the MSCs using the mirVana™ miRNA Isolation kit (Ambion, Austin, TX, USA) according to the manufacturer’s instructions and reverse transcribed into cDNA using the PrimeScript RT reagent kit (Takara Biochemicals). miR-130a/b was quantified using TaqMan^®^ MicroRNA Assay kits (Applied Biosystems) with U6 small nuclear RNA as an endogenous control. All PCR reactions were performed in triplicate.

### RNA interference

FOXM1 and control siRNA (scramble) were purchased from Santa Cruz Biotechnology, Inc. After the MSCs were transfected with FOXM1 or control siRNA using Lipofectamine™ 2000 (Invitrogen) as previously described by Wang *et al* (16), the cells were collected and processed for subsequent analysis by western blot analysis and quantitative PCR. The untreated MSCs served as the control group.

### SA-β-gal assay

MSC senescence was determined based on SA-β-gal activity, which was measured using a β-galactosidase staining kit (BioVision, Palo Alto, CA, USA) following the manufacturer’s instructions. Briefly, the treated MSCs were washed in PBS, fixed in 0.5 μl of fixative solution for 10–15 min at room temperature, and incubated with the staining solution mix overnight at 37°C. Green-stained cells and total cells were then counted under a microscope, and the percentage of β-galactosidase-positive and enlarged cells was calculated.

### miR-130b silencing by antagomiR-based treatment

Chemically modified antisense oligonucleotides (antagomiR-130b; RiboBio, Guangzhou, China) have been previously used to inhibit miR-130b expression in MSCs *in vitro* (17). Antagomir oligonucleotides were transfected into the MSCs at doses of 100 nmol/l using Lipofectamine 2000 (Invitrogen) according to the manufacturer’s instructions. As the controls, an unrelated negative control (NC; RiboBio) was transfected into the MSCs. Transfection efficiencies were determined by quantitative PCR, and the cells were processed for subsequent intervention or analysis on day 1 following transfection.

### Statistical analysis

The results are expressed as the means ± standard error of the mean (SEM). Statistical analysis, which included an unpaired Student’s t-test for comparisons between 2 groups and analysis of variance (ANOVA) with Bonferroni’s correction for multiple comparisons, was performed using SPSS software (version 14; SPSS Inc., Chicago, IL, USA). A P-value of <0.05 was considered to indicate a statistically significant difference.

## Results

### HG induces senescence in MSCs

**Figure 1:**
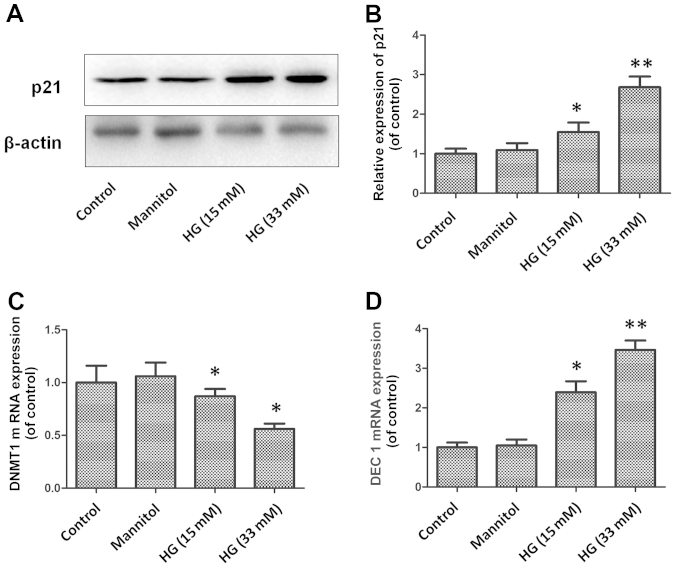
High glucose (HG) accelerates mesenchymal stem cell (MSC) senescence in a concentration-dependent manner. MSCs were exposed to various concentrations of HG for 24 h or to mannitol (33 mM), which served as a negative control with an osmotic pressure equal to that of 33 mM HG. (A and B) Detection of p21 expression in MSCs by western blot analysis. The representative bands and statistical bar diagram are shown. The data are presented as the means ± standard error of the mean (SEM); n=5 in each experiment. (C and D) DNA methyltransferase 1 (DNMT1) mRNA expression and differentiated embryo chondrocyte expressed gene 1 (DEC1) mRNA expression were examined by quantitative PCR; the results are illustrated in the bar diagram. The data are presented as the means ± SE from 5 independent experiments. ^*^P<0.05, ^**^P<0.01 vs. the control group.

To determine the effects of HG on MSC senescence, we performed a dose-effect experiment. Upregulated p21 expression, decreased DNMT1 mRNA expression and increased DEC1 mRNA expression were used as markers to evaluate cellular senescence. Incubation with HG for 24 h significantly enhanced p21 expression in the MSCs in a concentration-dependent manner by 155.3% (P<0.05) and 268.1% (P<0.01) in the 15 and 33 mM HG groups, respectively, compared with the control group; however, culture with mannitol at an osmotic pressure equal to that provided by 33 mM HG had no significant pro-senescence effects (P>0.05) on MSCs (Fig. 1A and B).

Quantitative PCR confirmed the effects of 33 mM HG on MSC senescence, as indicated by a 43.9% decrease (P<0.05) in DNMT1 mRNA expression and a 346.2% increase in DEC1 mRNA expression (P<0.01) compared with the control group (Fig. 1C and D). Analogously, the control, mannitol, did not have a notable effect on the mRNA expression of either DNMT1 or DEC1. Based on these results and those of a previous study (18), we selected the HG dose of 33 mM to establish an experimental model of MSC senescence in the subsequent experiments.

### ISDN treatment reduces HG-induced MSC senescence

**Figure 2:**
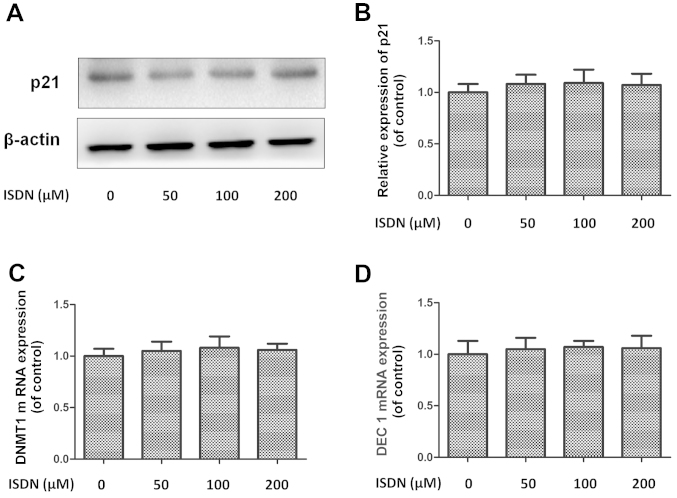
Isosorbide dinitrate (ISDN) does not have a notable effect on mesenchymal stem cell (MSCs) senescence. MSCs were cultured with ISDN at ISDN concentrations of 0 to 200 μM, and cellular senescence was compared between the groups. (A and B) p21 expression in MSCs was examined by western blot analysis; representative bands and a histogram are shown. The data are presented as the means ± standard error of the mean (SEM); n=5 in each group. (C and D) DNA methyltransferase 1 DNMT1 mRNA expression and differentiated embryo chondrocyte expressed gene 1 (DEC1) mRNA expression in MSCs was analyzed by quantitative PCR; the results are shown in the bar diagram. Data were obtained from 6 independent experiments, presented as the means ± SE, and compared with the control (untreated group; 0).

To observe the effect of ISDN pre-treatment on HG-induced MSC senescence, the MSCs were first cultured with ISDN at a concentration of 50, 100 or 200 μM for 24 h to determine the appropriate intervention dose of ISDN. At these concentrations, ISDN did not exert a significant effect on MSC senescence compared with the control group, as evidenced by p21 expression and DNMT1 and DEC1 mRNA expression (all P>0.05) (Fig. 2).

**Figure 3:**
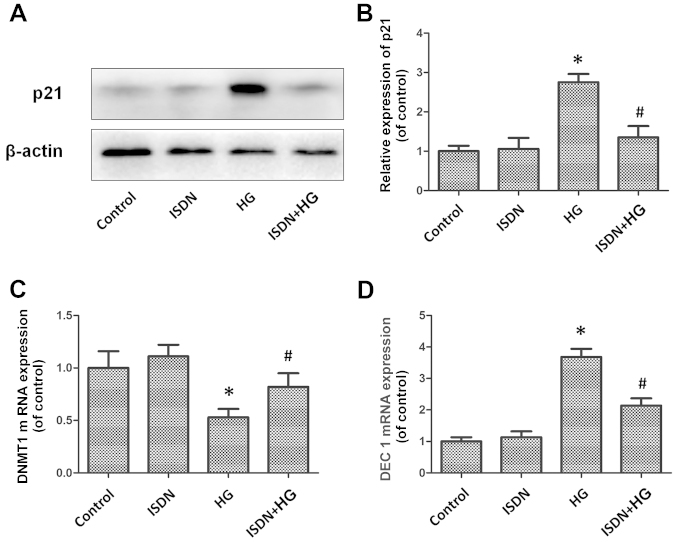
Isosorbide dinitrate (ISDN) preconditioning significantly attenuated the senescence of mesenchymal stem cells (MSCs) induced by high glucose (HG). MSCs were conditioned with or without ISDN (50 μM) for 6 h prior to HG treatment (33 mM) for an additional 24 h; cellular senescence was then investigated between the groups. (A and B) Cell aging was measured based on p21 expression, which was assessed by western blot analysis, and representative bands and a statistical histogram are shown. The results are presented as the means ± standard error of the mean (SEM); n=4 in each group. (C and D) Quantitative PCR was used to assess the mRNA expression of DNA methyltransferase 1 (DNMT1) and differentiated embryo chondrocyte expressed gene 1 (DEC1) in MSCs. The data are presented as the means ± SE and are shown in the histogram. Data were confirmed in 5 independent experiments. ^*^P<0.05 vs. control; ^#^P<0.05 vs. the HG group.

Subsequently, the MSCs were incubated with ISDN (50 μM) for 6 h and then cultured with 33 mM HG for an additional 24 h. Western blot analysis demonstrated that although incubation with 50 μM ISDN did not affect p21 expression [ISDN group vs. control (untreated) group, 1.06±0.28 vs. 1.00±0.14; P>0.05], treatment with 33 mM HG significantly upregulated p21 expression in the MSCs [HG group vs. control (untreated) group, 2.75±0.21 vs. 1.00±0.14; P<0.05], which was markedly attenuated after pre-conditioning with ISDN (ISDN + HG group vs. HG group, 1.35±0.29 vs. 2.75±0.21; P<0.05) (Fig. 3A and B). DNMT1 mRNA expression was significantly increased in the ISDN + HG group compared to the HG group (0.82±0.13 vs. 0.53±0.08, P<0.05) (Fig. 3C); DEC1 mRNA expression was decreased in the ISDN + HG group compared to the HG group (2.14±0.23 vs. 3.68±0.26, P<0.05) (Fig. 3D).

### The ERK/FOXM1 pathway is involved in the protective effects of ISDN against MSC senescence

**Figure 4:**
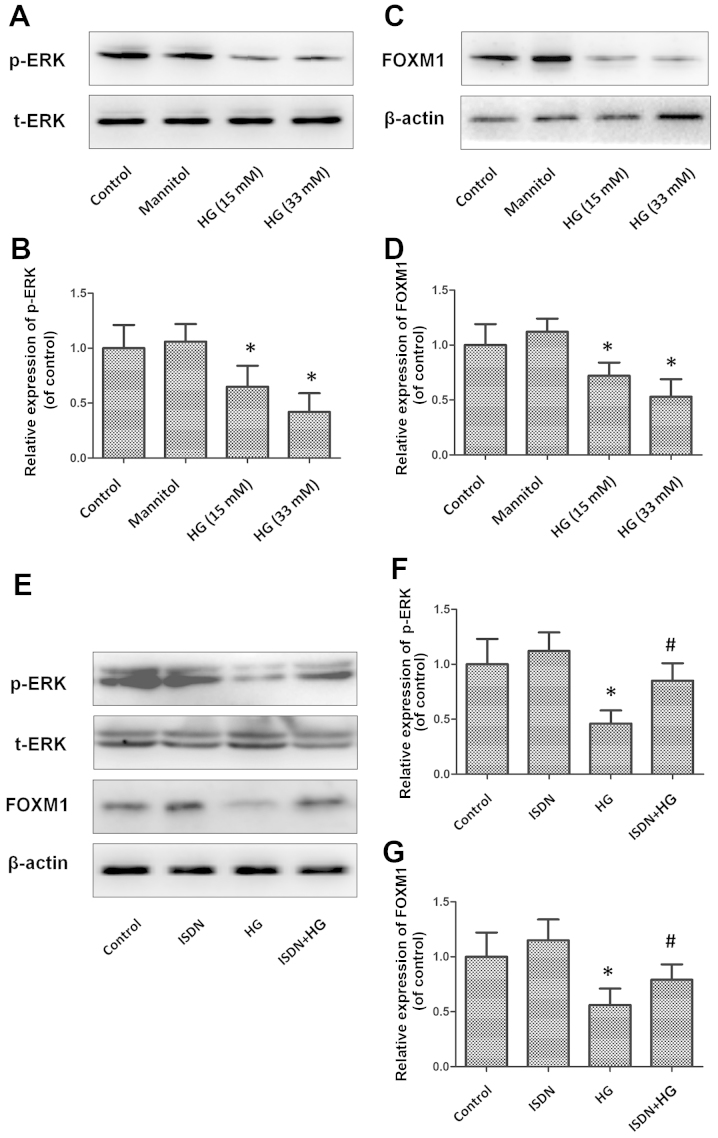
The ERK/FOXM1 pathway is affected by the induction of mesenchymal stem cell (MSC) senescence induced by high glucose (HG) and the protective effects of isosorbide dinitrate (ISDN). (A–D) MSCs were treated with HG at 15 mM or 33 mM or with mannitol (33 mM), which served as a negative control with an osmotic pressure equal to that of 33 mM HG. ERK phosphorylation and FOXM1 expression were evaluated by western blot, and representative bands are shown. The results are shown as the means ± standard error of the mean (SEM); n=3 per group. ^*^P<0.05 vs. the control. (E–G) MSCs were preconditioned with ISDN (50 μM) for 6 h, followed by HG treatment (33 mM) for an additional 24 h. Western blot analysis was used to measure the activity of the ERK/FOXM1 pathway: representative bands and a statistical bar diagram are shown. The data are presented as the means ± SEM; n=3 in each experiment. ^*^P<0.05 vs. the control group; ^#^P<0.05 vs. the HG group.

The ERK/FOXM1 pathway plays a key role in cellular senescence (19), and thus we investigated changes occurring in this pathway in MSCs following treatment with HG with or without pre-incubation with ISDN. HG induced the downregulation of ERK phosphorylation and FOXM1 expression in a dose-dependent manner (all P<0.05) (Fig. 4A–D). At a concentration of 33 mM, HG decreased ERK phosphorylation and FOXM1 expression by 53.8 and 43.9%, respectively, compared with the control (untreated) group (both P<0.05); however, the suppressive effects on the ERK/FOXM1 pathway were markedly reversed by pre-incubation with ISDN (ERK phosphorylation: ISDN + HG group vs. HG group, 0.85±0.16 vs. 0.46±0.12, P<0.05; FOXM1 expression: ISDN + HG group vs. HG group, 0.79±0.14 vs. 0.56±0.15, P<0.05) (Fig. 4E–G).

### Activation of the ERK/FOXM1 pathway mediates the effects of ISDN on MSC senescence

**Figure 5:**
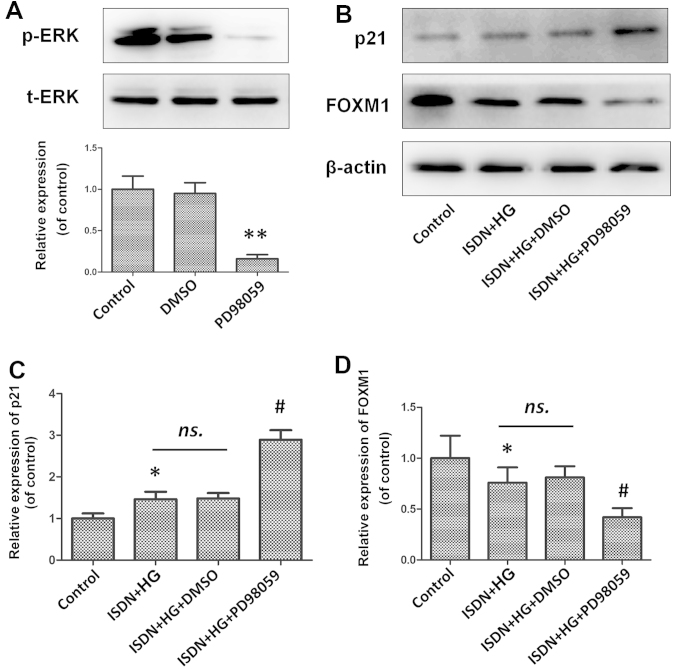
Inhibition of ERK phosphorylation abolishes the favourable effects of isosorbide dinitrate (ISDN) on mesenchymal stem cell (MSC) senescence. (A) MSCs were treated with PD98059 (ERK inhibitor; 20 μM) or DMSO (served as negative control) for 30 min, and ERK phosphorylation was then investigated by western blot analysis. Representative bands and a corresponding bar diagram are shown. ^**^P<0.01 vs. the control group. (B–D) MSCs were incubated with PD98059 (20 μM) or DMSO (served as negative control) for 30 min, followed by ISDN pre-culture for 6 h and high glucose (HG) treatment for an additional 24 h. Representative p21 and FOXM1 expression as determined by western blot analysis is shown, and the results are presented in the histogram. The data are presented as the means ± standard error of the mean (SEM); n=3 in each experiment. ^*^P<0.05 vs. the control group; ^#^P<0.05 vs. the ISDN + HG group; ns., not significant.

**Figure 6:**
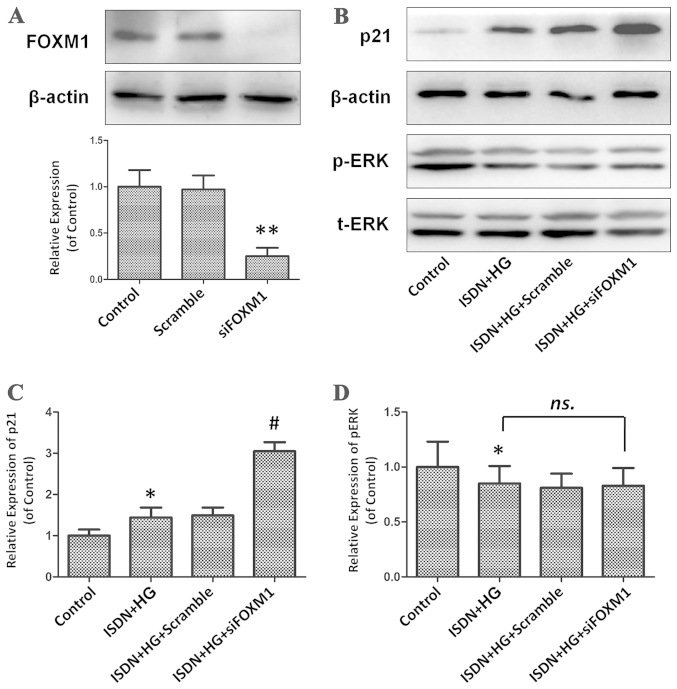
Knockdown of FOXM1 attenuates the beneficial effects of isosorbide dinitrate (ISDN) on mesenchymal stem cell (MSC) aging. (A) siRNA targeting FOXM1 or scrambled siRNA were transfected into the MSCs, and the representative bands and a histogram are shown with data presented as the means ± standard error of the mean (SEM) and confirmed in 3 independent experiments. ^**^P<0.01 vs. control group. (B–D) After the knockdown of FOXM1, MSCs were cultured with ISDN for 6 h and then treated with high glucose (HG) for another 24 h. p21 expression and ERK phosphorylation levels were determined by western blot analysis and are statistically demonstrated in the histogram. Data are presented as the means ± SE from 3 independent experiments. ^*^P<0.05 vs. the control group; ^#^P<0.05 vs. the ISDN + HG group;.ns., not significant.

To determine the role of the ERK/FOXM1 pathway in the effects of ISDN on HG-induced MSC senescence, PD98059, a specific inhibitor of the ERK pathway, and siRNA were used to inhibit the activity of the ERK/FOXM1 pathway (Figs. 5A and 6A). PD98059 effectively inhibited ERK phosphorylation by 83.6% compared with the control (untreated) group (P<0.01), which significantly inhibited the beneficial effects of ISDN on MSC senescence, as indicated by the upregulation of p21 expression in the ISDN + HG + PD98059 group compared with the ISDN + HG group (2.89±0.23 vs. 1.46±0.18, P<0.05) (Fig. 5B and C). Similarly, following the knockdown of FOXM1 in the MSCs by 74.8% using RNA interference, the protective effects of ISDN on HG-induced MSC senescence were significantly attenuated, as indicated by a comparison of the ISDN + HG + siFOXM1 and ISDN + HG groups; there was an increase in p21 expression in the ISDN + HG + siFOXM1 group compared with the ISDN + HG group (3.05±0.22 vs. 1.44±0.24, P<0.05) (Fig. 6B and C).

**Figure 7:**
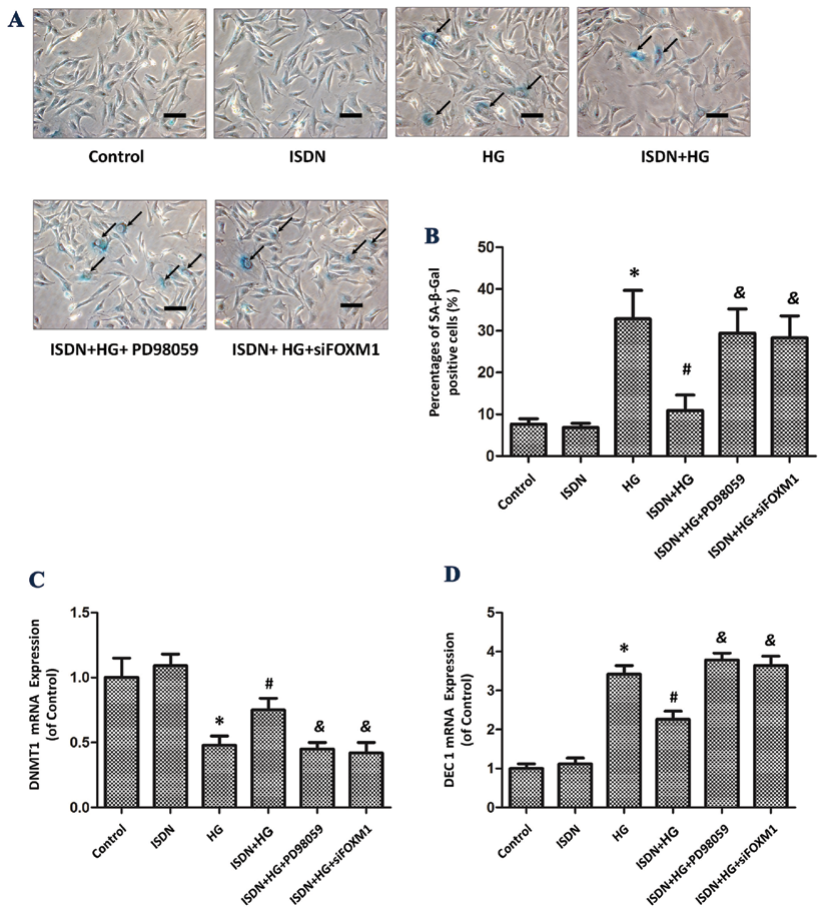
Isosorbide dinitrate (ISDN) pre-incubation delayed mesenchymal stem cell (MSC) senescence induced by high glucose (HG), which was mediated by the activation of the ERK/FOXM1 pathway. MSCs were incubated with PD98059 (ERK inhibitor; 20 μM) for 30 min prior to ISDN treatment (50 μM) for 6 h, followed by HG treatment (33 mM) for an additional 24 h. Alternatively, MSCs were treated with PD98059 or subjected to FOXM1 knockdown by siRNA and then treated with ISDN and HG. Senescence-associated β-galactosidase (SA-β-gal) activity and differentiated embryo chondrocyte expressed gene 1 (DEC1) and DNA methyltransferase 1 (DNMT1) mRNA expression were assessed to determine the cellular aging in each group. (A and B) Representative photomicrograph of the SA-β-gal assay is shown (scale bar, 20 μm). The percentage of β-galactosidase-positive cells (indicated by arrows) in each group was compared and is illustrated in the histogram; n=6 in each experiment. (C and D) Quantitative PCR analysis revealing the differences in DEC1 and DNA methyltransferase 1 (DNMT1) mRNA expression in each group. The data are presented as the means ± standard error of the mean (SEM); n=3 in each experiment. ^*^P<0.05 vs. the control group; ^#^P<0.05 vs. the HG group; ^&^P<0.05 vs. the ISDN + HG group.

A senescence-associated SA-β-gal assay was performed to verify the beneficial effects of ISDN on MSC senescence as revealed above. A higher percentage of senescent MSCs with a typically flattened and enlarged cell shape accompanied by SA-β-gal positivity was observed in the HG group compared to the control group (P<0.05) (Fig. 7A and B). Of note, pre-incubation with ISDN markedly decreased the HG-induced MSC senescence (ISDN + HG group vs. HG group, 10.9±3.72 vs. 32.8±6.83%; P<0.05). However, treatment with PD98059 significantly attenuated the effects of ISDN on MSC aging, indicated by the increased number of SA-β-gal-positive MSCs (29.4±5.81 vs. 10.9±3.72%, P<0.05) (Fig. 7A and B), as well as the decrease in DNMT1 mRNA expression (0.45±0.05 vs. 0.75±0.09, P<0.05) (Fig. 7C), and the increase in DEC1 mRNA expression (3.78±0.18 vs. 2.26±0.21, P<0.05) (Fig. 7D) in the ISDN + HG + PD98059 group compared with the ISDN + HG group. Accordingly, FOXM1 knockdown markedly abolished the effects of ISDN in MSC senescence, which was indicated by the increase in the number of MSCs with SA-β-gal positivity (28.3±5.25 vs. 10.9±3.72%, P<0.05) (Fig. 7A and B), as well as the decrease in DNMT1 mRNA expression (0.42±0.08 vs. 0.75±0.09, P<0.05) (Fig. 7C), and an increase in DEC1 mRNA expression (3.64±0.24 vs. 2.26±0.21, P<0.05) (Fig. 7D) in the ISDN + HG + siFOXM1 group compared with the ISDN + HG group.

### Upregulation of miR-130b is associated with the activation of the ERK/FOXM1 pathway

**Figure 8:**
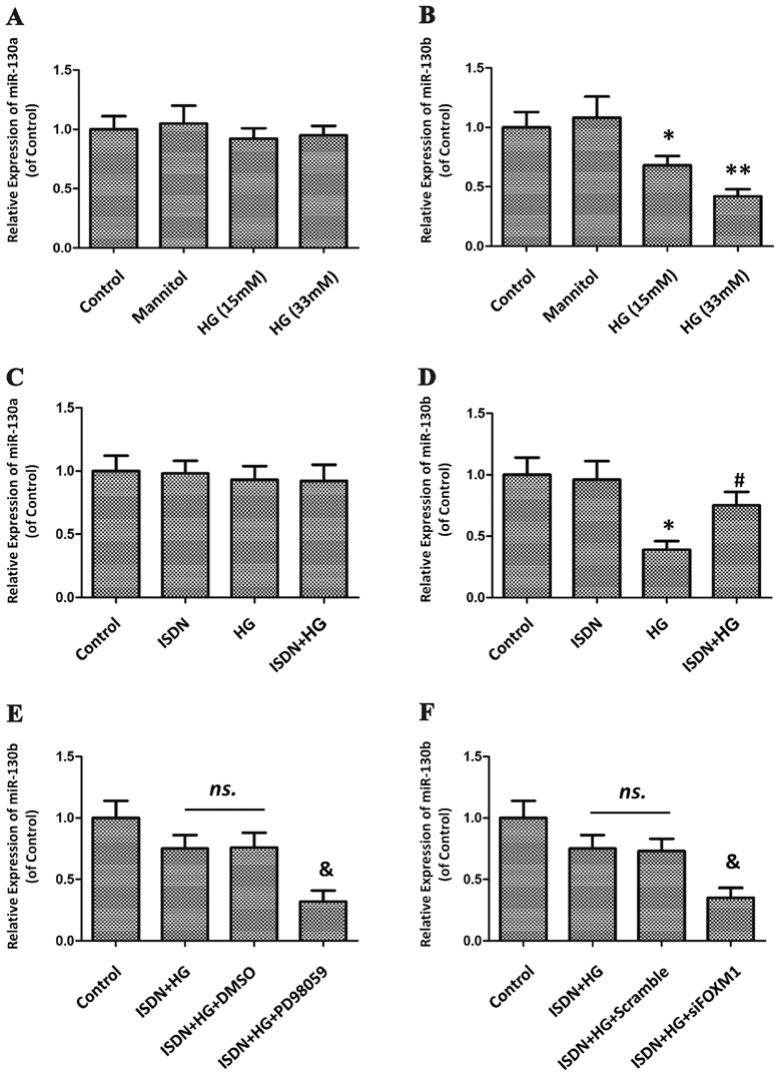
miR-130b, not miR-130a, is upregulated by the activation of the ERK/FOXM1 cascade during the protection of high glucose (HG)-induced mesenchymal stem cell (MSC) senescence by isosorbide dinitrate (ISDN). (A and B) MSCs were exposed to HG at progressively increasing concentrations for 24 h or to mannitol (33 mM), which served as a negative control with an osmotic pressure equal to that of 33 mM HG. The expression of the miR-130 family, including miR-130a and -130b, was determined by quantitative PCR and is shown in the histogram. The data are presented as the means ± standard error of the mean (SEM); n=3 in each experiment. ^*^P<0.05, ^**^P<0.01 vs. the control group. (C and D) MSCs were incubated with or without ISDN (50 μM) for 6 h, followed by HG treatment (33 mM) for an additional 24 h. The expression of miR-130a/b was analyzed by quantitative PCR, and the results are presented in the histogram. The data are presented as the means ± standard error of the mean (SEM); n=3 in each experiment. ^*^P<0.05 vs. the control group; ^#^P<0.05 vs. the HG group. (E and F) MSCs were cultured with PD98059 (20 μM) for 30 min prior to ISDN treatment (50 μM, 6 h), followed by HG treatment (33 mM, 24 h). Alternatively, MSCs were treated with PD98059 or subjected to FOXM1 knockdown by siRNA and then treated with ISDN and HG. miR-130b expression was evaluated by quantitative PCR, and the results are shown in the bar diagram. The data are presented as the means ± SEM; n=3 in each experiment. ns., not significant; ^&^P<0.05 vs. the ISDN + HG group.

The key regulatory role of the miR-130 family, including miR-130a and miR-130b, in cellular growth arrest has previously been demonstrated (14). In this study, we therefore examined the potential involvement of miR-130a and miR-130b in the response to HG treatment and the effects of ISDN. HG gradually suppressed miR-130b expression (all P<0.05) in a concentration-dependent manner, but had no detectable effect on miR-130a expression (all P>0.05) (Fig. 8A and B). Pre-treatment with ISDN reversed the decrease in miR-130b expression induced by HG stimulation (ISDN + HG group vs. HG group, 0.75±0.11 vs. 0.39±0.07; P<0.05), but had no apparent effect on miR-130a expression (ISDN + HG group vs. HG group, 0.92±0.13 vs. 0.93±0.11; P>0.05) (Fig. 8C and D). Furthermore, inhibition of the ERK/FOXM1 pathway by either PD98059 or FOXM1 siRNA substantially abolished the recovery of miR-130b expression following pre-treatment with ISDN (ISDN + HG + PD98059 group vs. ISDN + HG group: 0.32±0.09 vs. 0.75±0.11, P<0.05; ISDN + HG + siFOXM1 group vs. ISDN + HG group: 0.35±0.08 vs. 0.75±0.11, P<0.05) (Fig. 8E and F).

### miR-130b is the key mediator of the effects of ISDN on MSC senescence

**Figure 9:**
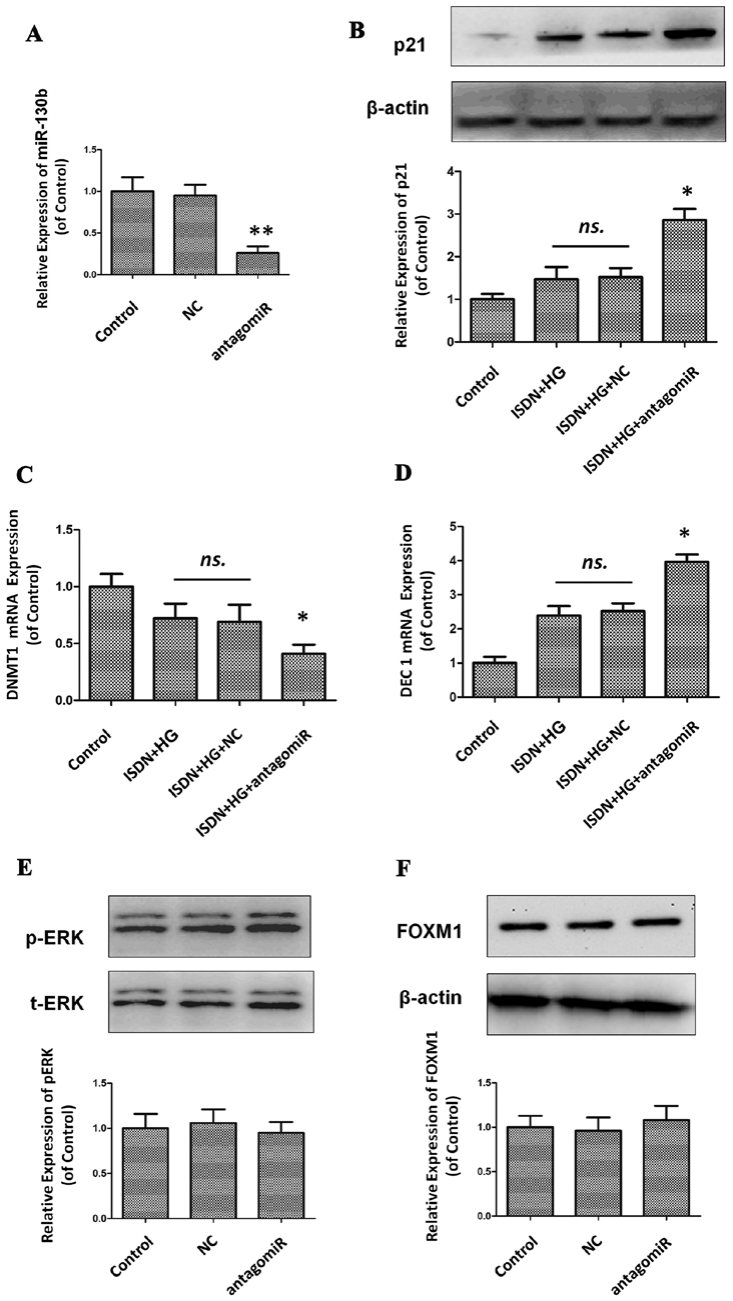
Downregulation of miR-130b abolishes the beneficial effects of isosorbide dinitrate (ISDN) on mesenchymal stem cell (MSC) aging. (A) The miR-130b level was quantified by quantitative PCR. The expression value of the control group was designated 1, and the levels of other groups were calibrated to this value. Data was presented as the means ± standard error of the mean (SEM) and confirmed in 5 independent experiments. ^**^P<0.01 vs. control group. (B–D) Follwing the downregulation of miR-130b, MSCs were incubated with ISDN for 6 h and then exposed to high glucose (HG) for an additional 24 h. p21 expression was determined by western blot analysis. Quantitative PCR detection was further performed to reveal the differences in differentiated embryo chondrocyte expressed gene 1 (DEC1) and DNA methyltransferase 1 (DNMT1) mRNA expression in each group. The data are presented as the means ± SEM from 5 independent experiments. ^*^P<0.05 vs. the ISDN + HG group; ns., not significant. (E–F) ERK phosphorylation and FOXM1 expression were determined following the suppression of miR-130b. Representative western blots are shown and the results are presented in the bar diagram as the means ± SEM from 5 independent experiments. NC, negative control.

To determine whether miR-130b plays a key role in the protective effects of ISDN against MSC senescence, miR-130b was knocked down using antagomiR transfection and senescence-associated assays were then performed. It was found that after miR-130b expression was effectively downregulated by 74.3% compared to the control (Fig. 9A), the beneficial effects of ISDN on MSC senescence were markedly abolished, which was confirmed by the analysis of p21 expression (1.47±0.29 vs. 2.86±0.26, P<0.05), DNMT1 mRNA expression (0.72±0.13 vs. 0.41±0.08, P<0.05) and DEC1 mRNA expression (2.39±0.28 vs. 3.97±0.21, P<0.05) (Fig. 9B–D) when comparing the ISDN + HG group with the ISDN + HG + antagomiR group.

ERK phosphorylation and FOXM1 activity were examined following the downregulation of miR-130b, indicating that there were no signficant changes between the control group and the antagomiR group, both in phosphorylated ERK (1.00±0.16 vs. 0.95±0.12, P>0.05) and in FOXM1 expression (1.00±0.13 vs. 1.08±0.16, P>0.05) (Fig. 9E and F).

## Discussion

This study aimed to investigate the effects of ISDN on HG-induced MSC senescence, demonstrating that ISDN preconditioning decreases SA-β-Gal activity in MSCs, decreases the expression of p21 protein and DEC1 mRNA, and increases the expression of DNMT1 mRNA, consistent with our original assumption that ISDN inhibits MSC aging triggered by HG. The activation of the ERK/FOXM1 pathway plays a pivotal role in this pathophysiological process, as evidenced by the attenuation of the favourable effects of ISDN by either the inhibition of ERK phosphorylation or the knockdown of FOXM1 in the MSCs. As a senescence-associated modulator, miRNA-130b was identified to function downstream of the ERK/FOXM1 pathway, collectively contributing to the protective effects of ISDN against MSC aging.

Diabetic patients frequently suffer from micro- or macrovascular abnormalities, including neuropathy, nephropathy, retinopathy and accelerated ischemic heart diseases, which mobilize MSCs to repair and regenerate damaged tissue. Although there is a complex condition which induces MSC growth arrest, it is undoubtedly considered that HG is the primary trigger. Accordingly, HG incubation, not oxidative stress or ultraviolet B radiation (UVB) treatment, was developed to establish a cellular senescence model in MSCs, which may better simulate the true pathophysiological state in diabetes mellitus (DM) (20), and may have great practical implications for the treatment of patients with DM accompanied by its various complications.

Organic nitrates are one of the most common types of drugs used for the long-term treatment of acute coronary syndrome and acute or chronic congestive heart failure, as well as coronary heart diseases (21). The release of NO is the main function of ISDN (22) and leads to the activation of soluble guanylate cyclase and the reduction of the intracellular calcium content, subsequently resulting in the relaxation of target cells, primarily the vascular smooth muscle (23). However, a comprehensive understanding of the mechanisms of action of organic nitrates, particularly other non-hemodynamic effects on other various cells, has not yet been established (24). Given the critical role of MSCs in cardiovascular diseases, we wished to determine whether treatment with ISDN affects the biological characteristics of MSCs.

Senescent MSCs exhibit a significantly reduced ability to self-renew, growth is terminated at an early stage at approximately 40–50 population doublings, and a decline in differentiation potential and proliferation rate is observed (25). Furthermore, senescent MSCs provide a weak protective effect due to the decreased activity of repair and antioxidant enzymes in aged MSCs, resulting in the unsatisfactory effects of MSC-based treatments for tissue repair (26). Several pathophysiological states, such as HG and oxidative stress, are considered critical contributors to cell senescence, as well as predominant risks or accompanying factors in cardiac ischemia, which is regularly treated with organic nitrates (27). Therefore, our identification of ISDN as a modulator of MSC senescence may provide insight into the protective mechanisms of organic nitrates in cardiovascular diseases. However, an investigation using DM models *in vivo*, comparing MSC senescence before and after receiving organic nitrate treatment, is required in order to further confirm the *in vitro* above findings.

The ERK/FOXM1 pathway is involved in cell cycle regulation and senescence modulation (12,13). However, whether it accelerates or delays cell aging is dependent on the cell type and pathophysiological conditions. Ling *et al* (28) observed that the activated ERK phosphorylation and upregulated FOXM1 expression were essential for the protective effects of low-power laser irradiation against NIH3T3 cell senescence induced by UVB. By contrast, Wang *et al* (19) demonstrated that the N terminus of ASPP2 binds to Ras, enhancing Ras/Raf/MEK/ERK activation to facilitate oncogene-induced senescence; Zeng *et al* (29) discovered that FOXM1 is upregulated in gastric cancer and that its inhibition led to termination of the cell cycle at the G0/G1 phase, partially dependent on p27 kip1. In light of these results, we examined changes in the activities of ERK and FOXM1 in HG-treated MSCs and in response to the protective effects of ISDN. HG significantly suppressed ERK phosphorylation and FOXM1 expression, which were both attenuated by ISDN preconditioning. Furthermore, the inhibition of ERK phosphorylation or the knockdown of FOXM1 attenuated the favourable effects of ISDN on MSC aging, indicating that ERK and FOXM1 are key mediators of the effects of ISDN. While the inhibition of ERK phosphorylation markedly decreased the upregulation of FOXM1, the downregulation of FOXM1 did not exert a significant effect on ERK activity, revealing the regulatory association between these two proteins.

Considering the crucial function of miRNAs in cell biology, we also investigated the senescence-associated miRNAs based on the review by Abdelmohsen *et al* (14), including those that elicit their actions through the pRB/p16 pathway (miR-106, miR-130 and miR-24) or the p53 pathway (miR-34), affect senescence-associated secretory phenotypes (SASP) (miR-146) and modulate other senescence regulatory proteins (miR-29, miR-30 and miR-519). As demonstrated by quantitative PCR (data not shown), we determined that only miR-130b and not miR-130a is involved in HG-induced MSC aging, as well as the subsequent ISDN protective effects. Subsequently, we examined miR-130b expression following the suppression of the ERK/FOXM1 cascade and hence deduced that miR-130b functions downstream of this pathway. Based on previous studies, it has been demonstrated that miR-130b inhibits the expression of p21, which is regarded to be antagonistic to p53/p21 pathway activity, leading to a recovery of growth arrest in proliferative cells (30,31). Borgdorff *et al* (32) established an important role for the cyclin-dependent kinase (CDK) inhibitor, p21 (Waf1/Cip1), in the growth control of human mammary epithelial cells (HMECs) and analyzed the repertoire of miRNAs that modulate the activity of this tumor suppressor, demonstrating that miR-130b reduced p21 expression and in turn blocked Ras (G12V)-induced senescence. They further revealed that the overexpression of miR-130b rescued HMECs from Ras-induced senescence through the prevention of Ras-induced upregulation of p21, verifying the key role of miR-130b during proliferative cell senescence, which powerfully supported the underlying mechanisms found in our study. Considering that the regulation of miRNAs differs depending on cell type, we wished to determine whether the inhibition of miR-130b reduces the protective effects of ISDN on MSC senescence. We therefore silenced miR-130b expression in MSCs, revealing that miR-130b downregulation markedly attenuated the advantageous effects of ISDN on MSC senescence, which was indicated by the detection of p21 expression and the mRNA expression of DNMT1 and DEC1. Furthermore, we revealed that both ERK phosphorylation and FOXM1 expression were not significantly affected by miR-130b suppression, together with our prior finding that the suppression of either ERK phosphorylation or FOXM1 expression markedly reduced miR-130b expression, collectively indicating that miR-130b resides downstream of the ERK/FOXM1 pathway. However, the exploration of the role of miR-130b in MSC senescence in the current study is preliminary, and further research focusing on the presumed target of miR-130b is required, including the prediction of informatics algorithm, luciferase report assay and functional complementary experiments. We aim to resolve these issues in our future research, which may broaden our understanding of the association between MSC senescence and miRNA modulation.

Support for our hypothesis can also be found in previous studies of the pharmacological function of organic nitrates on cell senescence or growth arrest. Huang *et al* (33) demonstrated that nitroglycerine (NTG) within a concentration range of 0.1–10 μmol/l notably increased BrdU incorporation into human MSCs in a dose-dependent manner, suggesting that NTG enhances the cell growth rate and activity. The authors further determined that the promotion of NO release in MSCs by NTG was a key mechanism underlying this effect, suggesting that organic nitrate stimulates NO production in MSCs, leading to the increased cell viability. Moreover, the use of organic nitrates in phytophysiology (34) has indicated that nitrate limitation or starvation markedly induces leaf senescence based on a combined ^15^N tracing/proteomics analysis (35) and SAG12-transgenic plant construction (36). In addition, it has been reported that the long-term inhibition of NO synthase by L-arginine analogues, such as N(ω)-nitro-l-arginine methyl ester (L-NAME), accelerates vascular senescence *in vitro* and systemic hypertension and arteriosclerosis *in vivo* (37). Furthermore, it has been demonstrated that pre-treatment with various Chinese traditional medicines promotes the renovation of H_2_O_2_-stimulated senescence in human umbilical endothelial cells via nitrate production (38); and that elevated NO production, which can also be promoted by organic nitrates, in MSCs overexpressing eNOS enhances cellular activity and migration through the upregulation of stromal cell-derived factor-1α (39), which conformably evidenced the significance and reasonability of our novel finding.

In conclusion, to the best of our knowledge, the present study provides evidence that ISDN exerts protective effects agaisnt HG-induced MSC senescence, and that the underlying mechanisms involve the activation of the ERK/FOXM1 pathway and the upregulation of miR-130b. The data presented in this study may prove to be of clinical value for patients with DM accompanied by diverse clinical complications.
